# Highlights of Precision Medicine, Genetics, Epigenetics and Artificial Intelligence in Pompe Disease

**DOI:** 10.3390/ijms26020757

**Published:** 2025-01-17

**Authors:** Marta Moschetti, Marika Venezia, Miriam Giacomarra, Emanuela Maria Marsana, Carmela Zizzo, Giulia Duro, Annalisa D’Errico, Paolo Colomba, Giovanni Duro

**Affiliations:** 1 Institute for Biomedical Research and Innovation (IRIB), National Research Council (CNR), 90146 Palermo, Italy; marika.venezia@irib.cnr.it (M.V.); miriam.giacomarra@irib.cnr.it (M.G.); emanuelamaria.marsana@irib.cnr.it (E.M.M.); carmela.zizzo@irib.cnr.it (C.Z.); annalisa.derrico@irib.cnr.it (A.D.); paolo.colomba@irib.cnr.it (P.C.); giovanni.duro@irib.cnr.it (G.D.); 2 Internal Medicine, Ospedale Cattinara, 34149 Trieste, Italy; giulia.duro@libero.it

**Keywords:** Pompe disease, glycogen metabolism, skeletal muscle disease, epigenetics, artificial intelligence (AI)

## Abstract

Pompe disease is a neuromuscular disorder caused by a deficiency of the enzyme acid alpha-glucosidase (*GAA*), which leads to lysosomal glycogen accumulation and progressive development of muscle weakness. Two distinct isoforms have been identified. In the infantile form, the weakness is often severe and leads to motor difficulties from the first few months of life. In adult patients, the progression is slower but can still lead to significant loss of mobility. The current inherent difficulties of the disease lie in both early diagnosis and the use of biomarkers. Given that this is a multifactorial disease, a number of components may exert an influence on the disease process; from the degree of pre-ERT (enzyme replacement therapy) muscle damage to the damaged autophagic system and the different pathways involved. What methodology should be employed to study the complex characteristics of Pompe disease? Our approach relies on the application of genetic and epigenetic knowledge, with a progression from proteomics to transcriptomics. It is also becoming increasingly evident that artificial intelligence is a significant area of interest. The objective of this study is to conduct a comprehensive review of the existing literature on the known data and complications associated with the disease in patients with disorders attributed to Pompe disease.

## 1. Introduction

**Figure 1:**
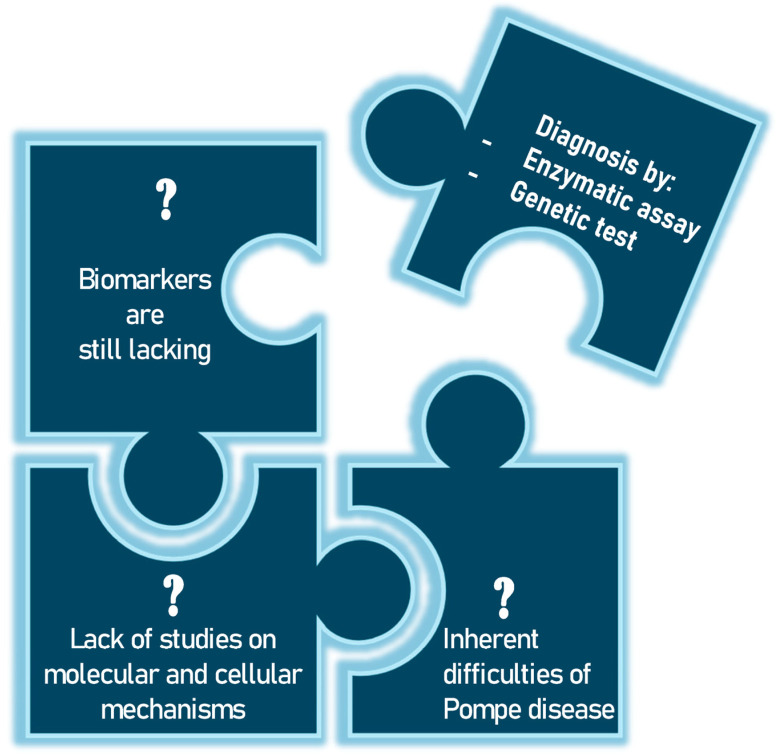
The unsolved problems of Pompe disease have so far only been addressed by biochemical and genetic tests.

Pompe disease (PD), also known as glycogenosis type II, is a rare inherited metabolic disorder caused by a deficiency of the enzyme acid alpha-glucosidase (*GAA* enzyme) [1]. This enzyme is essential for the degradation of glycogen in lysosomes, and its deficiency leads to an accumulation in these cellular structures, particularly in skeletal muscle, smooth muscle, the heart and, to a lesser extent, the central nervous system [2,3]. A cascade of pathological events ensues, including the disruption of cellular homeostasis, activation of inflammatory pathways and cell apoptosis (programmed cell death). Depending on the severity of the enzyme deficiency, which in turn depends on the type of mutation in the *GAA* gene, the clinical manifestations of PD vary widely. The classic infantile form of PD (IOPD) is the most severe and manifests in the first few months of life. Affected infants present with severe hypertrophic cardiomyopathy, generalized muscle hypotonia, respiratory distress and delayed motor development [4]. If left untreated, this form is usually fatal within the first year of life due to cardiac or respiratory failure. Late-onset forms (LOPD) include those that begin in childhood from the age of two, in adolescence or in adulthood. Patients with these forms have progressive muscle weakness, particularly in the shoulder and pelvic girdle muscles, which can lead to difficulty walking and respiratory failure. Cardiomyopathy is less common in the late forms, but may be present in some cases [5]. These symptomatic manifestations are a wake-up call for the clinician, who initiates an investigation to diagnose the disease. Biochemical tests are performed on a suspect sample and, if this shows reduced or absent enzyme activity, genetic tests specific to the *GAA* gene are performed. The biochemical test can be performed on different types of samples, including dried blood on filter paper (DBS), which allows non-invasive initial screening, particularly useful in newborn babies, while the Sanger sequencing method is the method of choice for studying the *GAA* gene located on chromosome 17 [6]. But, given the rarity of the disease, numerous questions remain to be answered (Figure 1).

Unfortunately, biomarkers are still lacking, but the scientific community is continuing its research to enable early diagnosis and to find the key that may be useful not only for the detection of PD but also for monitoring disease progression after treatment [7]. Although very significant progress has been made in recent years in developing new projects and improving neonatal screening program and tools for more accurate diagnosis and patient follow-up, there is an unmet need to further understand the molecular mechanisms underlying disease progression. In this context, the study of molecular and cellular mechanisms and pathophysiology could be a crucial method to analyze the overall effect of lysosomal dysfunction and to investigate its possible relationship with clinical evolution and response to therapies. This could lead to the identification of new biomarkers potentially relevant to this disease. For example, Nuria Gómez-Cebrián’s group aims to highlight their key findings from recent studies based on omics approaches, with a particular focus on the clinical role of specific metabolic phenotypes associated with different subgroups of PD patients [8]. Finding a tool to help clinicians diagnose PD at an early stage is very important. Although muscle biopsy is a direct method of assessing the presence of this accumulation, its invasiveness makes it less desirable than other diagnostic approaches. For this reason, the scientific community continues to search for other candidates. In their article, Rafael Calais Gaspar et al. explore the hypothesis that small molecule inhibition of the enzyme glycogen synthase I (GYS1) can reduce muscle glycogen content and improve metabolic dysregulation in a mouse model of PD [9]. Jianwei Ren et al. highlight the testing of 1,5-anhydroglucitol (1,5-AG), 6-α-D-glucopyranosyl-maltotriose (Glc4) and maltotetraose (M4). The work supports the exploration of the potential of maltotetraose as a biomarker for PD [10]. In recent years, differential mobility spectrometry (DMS) has also become an effective analytical technique, with high selectivity and specificity [11]. Jianwei Ren and colleagues highlight this aspect to develop an efficient analytical method for the two urinary tetrasaccharide metabolites using DMS [12]. With the advent of advanced genomic and proteomic techniques, efforts are being made to initiate projects to study the pathophysiology of PD. However, to date, no new potential biomarkers have been identified. New investigations can be undertaken in PD, and many topics could provide information not only on diagnosis, but also on disease severity and response to treatment. For example, there could be a greater focus on molecular mechanisms of damage, such as oxidative stress. Reactive oxygen species (ROS) damage cell membranes and proteins, impairing muscle function or mitochondrial damage [13,14]. Mitochondria, which are crucial for ATP production, can become dysfunctional, reducing the muscle’s energy capacity [15]. Another very interesting area could be the study of changes in calcium metabolism. Intracellular calcium is crucial for contraction; imbalances can cause cellular damage and functional changes. Consequently, the investigation of genetic polymorphisms that impact muscle structure and metabolism can be employed to examine the genotype–phenotype correlation in PD.

A deeper comprehension of the potential influence of a plethora of “exercise genes” on the severity and progression of muscle dysfunction in PD could prove to be a significant advancement in our understanding of the disease, potentially leading to earlier diagnosis. An illustrative example is the ACE gene, which encodes for the angiotensin-converting enzyme. This gene has been the subject of study in relation to PD due to its potential influence on disease progression. Polymorphisms in the ACE gene, in particular the presence or absence of an insertion/deletion (I/D) fragment, have been demonstrated to impact circulating levels of angiotensin-converting enzyme (ACE) and the inflammatory response in muscle tissue [16,17]. In studies of PD, patients with the DD variant of the ACE gene (the “deletion” form) tended to have a more severe disease course, with a greater susceptibility to muscle damage [18]. This effect may be related to increased tissue ACE activity, which triggers inflammatory responses and promotes tissue remodeling, exacerbating the progression of muscle symptoms [16]. However, it remains to be determined whether the DD genotype of the ACE gene also affects the response to enzyme replacement therapy, which is the main treatment for PD [19].

**Figure 2:**
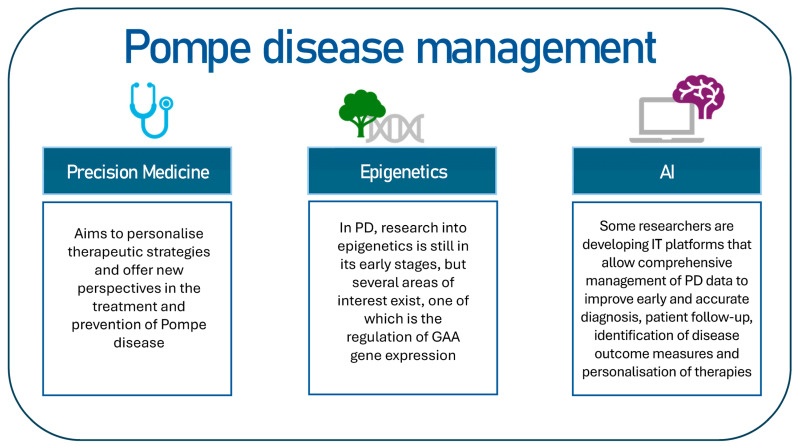
The following proposal constitutes a hypothetical scheme on how to intervene in the management of Pompe disease.

The objective of this review is to identify potential key topics for the management of Pompe disease (Figure 2). This review will examine investigative perspectives, including precision medicine and the potential of artificial intelligence in the context of PD.

## 2. Precision Medicine: Inherent Difficulties of the Disease

Advances in early diagnosis and the introduction of enzyme replacement therapy (ERT) have significantly improved the prognosis of patients, particularly in the infantile form (IOPD) [20]. However, many challenges remain in the long-term management of the disease. Research continues to focus on new therapeutic approaches and the optimization of existing therapies with the aim of developing optimal drugs to further improve the quality of life of affected patients.

The complexity of Pompe disease stems from a poor understanding of its pathophysiology and limited knowledge of its natural history. The inherent heterogeneity of PD, together with the difficulty of defining clinical endpoints, poses significant challenges [21]. Precision medicine, which is based on the genetic, environmental and lifestyle characteristics of the individual patient, aims to personalize therapeutic strategies and offers new perspectives in the treatment and prevention of PD. Chanchala Kaddi et al. created a model to represent the key elements of PD pathophysiology [22]. The QSP model was used to create digital replicas of each IOPD patient enrolled in the aval-glucosidase alpha clinical trial, taking into account disease-specific burden, demographic characteristics and previous treatment history. This virtual cohort was used to enhance clinical observations by simulating and comparing tissue glycogen and urinary Hex4 levels after treatment with aval-glucosidase alpha compared with standard care [23].

Elucidating the mechanism by which stored compounds affect cell function is the basis not only for understanding the pathophysiology of lysosomal storage diseases but also for targeted intervention [22]. The accumulation of non-degraded substrates interferes with several mechanisms, including the activation of receptors by non-physiological ligands, modulation of receptor responses and signaling cascades, activation of an inflammatory response, impairment of contractile function, alteration of intracellular vesicle trafficking and disruption of autophagy-related mechanisms [24]. Understanding these mechanisms is crucial because each event in the PD pathogenetic cascade represents a potential target for therapy and thus for precision medicine. It is therefore possible that a variety of therapeutic approaches, based on different strategies and rationales, each directed at a different therapeutic target, may be used individually or in combination to treat PD.

Another area where precision medicine can intervene is in the study of genetics. To identify the disease, it is necessary to identify specific mutations in the *GAA* gene that may be associated with disease severity [25]. With next-generation DNA sequencing (NGS) technologies, it is possible to quickly and accurately analyze the entire exome, enabling detailed and personalized genetic diagnosis. A discussion on the clinical implications of NGS in PD could emphasize its role in expediting accurate diagnoses, guiding targeted treatments and informing genetic counseling. Many papers highlight the clinical utility of the NGS technique in patients with suspected muscle disorders and its potential in facilitating the diagnosis of patients showing non-specific muscle weakness or atypical phenotypes [26,27,28]. A seminal study has revealed that, in a Taiwanese cohort of patients diagnosed with CRIM-positive (cross-reactive immunological material positive) IOPD by neonatal screening, the commencement of ERT within the first month of life resulted in enhanced long-term outcomes, encompassing independent ambulation and ventilator-free survival. Notwithstanding the demonstration of substantial benefits from early treatment, the study did not encompass the most severe phenotype: CRIM-negative (cross-reactive immunological material negative) IOPD. Li et al. investigated the benefits of early ERT in conjunction with rituximab, methotrexate and IVIG in patients with CRIM-negative IOPD, and compared the outcomes with those of CRIM-negative patients treated beyond 4 weeks of age [29]. The study population comprised 20 patients with CRIM-negative IOPD, who were grouped according to the age at which they received ERT: early (before 4 weeks of age), intermediate and late treatment groups [29]. Patients treated early demonstrated superior clinical outcomes and exhibited a lower reliance on respiratory or feeding assistance compared with those treated at an age greater than 4 weeks [29].

Some mutations are associated with more severe forms, such as those with childhood onset, while others are associated with milder late-onset forms. The disease can therefore affect people of all ages, from babies to adults, with symptoms of varying severity. This variability makes diagnosis particularly complex, and the overlap of its symptoms with those of other neuromuscular diseases can lead to misdiagnosis. Indeed, one of the main inherent difficulties of PD is its extreme clinical heterogeneity and, consequently, its correct diagnosis [30]. In order to establish a targeted therapy, it is important to analyze the cross-reactivity status to immunological materials: CRIM+ and CRIM− [31]. Patients with early-onset lysosomal storage diseases are ideal candidates for prenatal therapy because organ damage begins in utero. Cohen et al. reported the safety and efficacy results of in utero ERT in a CRIM-negative fetus with infantile-onset PD [32].

It is also crucial to consider the inherent difficulties associated with a muscle-specific therapeutic approach [25]. The challenges associated with PD extend beyond the medical domain and have a significant impact on the daily lives of patients and their families [33]. The impact on quality of life is a key factor in determining the course of the disease. Patients with PD, particularly those with late-onset PD, frequently experience progressive muscle weakness and an increasing reliance on respiratory support and assisted mobility. This physical determination has a significant impact on quality of life, limiting patients’ capacity to work, engage in social activities and pursue personal interests. In addition to medical treatment, psychological support is essential for the management of anxiety, depression and stress associated with a chronic and progressive disease. Patients and their families frequently experience uncertainty regarding disease progression and treatment response, which can precipitate considerable emotional distress. Another crucial aspect that necessitates attention is the socio-economic challenge. PD is a costly condition to treat and necessitates a long-term financial commitment, which can present an economic challenge for many families. Furthermore, the necessity for continuous specialized care may restrict access to employment and education, creating additional economic and social pressures [34].

## 3. Genetics in Pompe Disease

PD is caused by a genetic deficiency of the lysosomal enzyme alpha-glucosidase acid (*GAA*), which leads to progressive muscle weakness and respiratory failure [35]. To make a diagnosis, it is important to start by studying the *GAA* gene to identify disease-causing mutations. Sanger sequencing is becoming a powerful tool for clinical diagnosis to analyze the entire gene sequence and determine the variants present. This test is useful not only to confirm the diagnosis but also to determine the prognosis, as genetic variants influence the course of the disease [36].

The genetic underpinnings of lysosomal dysfunction in PD primarily involve mutations in the *GAA* gene, which encodes the lysosomal enzyme acid alpha-glucosidase (*GAA*). Pathogenic variants in *GAA* result in deficient enzymatic activity, leading to lysosomal glycogen accumulation, a hallmark of PD. This accumulation triggers a cascade of downstream effects, including secondary autophagic impairment [37]. Emerging evidence suggests that this autophagic dysfunction may be influenced by genetic variants in autophagy-related genes, such as those encoding proteins in the mTOR signaling pathway and the autophagy machinery [38]. In parallel, genetic contributions to mitochondrial dysfunction observed in PD have been linked to cross-talk between lysosomal and mitochondrial pathways. Variants in genes regulating mitochondrial dynamics, such as OPA1 and MFN2, may exacerbate metabolic perturbations [39]. Furthermore, dysregulated calcium homeostasis in PD has been associated with altered expression of calcium-regulating genes, including RYR1 and CASQ1, which are critical for sarcoplasmic reticulum function and muscle contractility [40]. Understanding the genetic landscape of PD and related skeletal muscle diseases not only deepens our knowledge of disease pathogenesis but also identifies potential therapeutic targets [41]. Gene therapy approaches, such as *GAA* replacement or modulation of autophagy-related genes, are promising strategies to restore cellular homeostasis [42].

**Figure 3:**
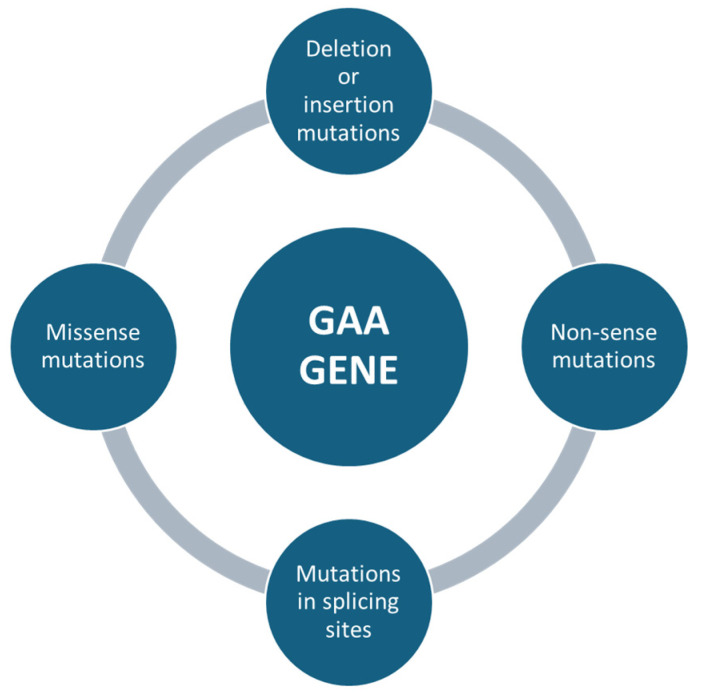
The following summary outlines the primary genetic mutations in the *GAA* gene that have been linked to Pompe disease. The mutations are listed in order to emphasize the most significant alterations.

Mutations affecting the *GAA* gene can be of different types, e.g., missense mutations, which are single nucleotide changes that result in the substitution of an amino acid in the protein sequence (Figure 3). Depending on the severity of the mutation, it can lead to a partial loss of enzyme activity (in late forms of the disease) or almost a complete loss of the enzyme (in severe childhood forms) [43]. Non-sense mutations, in which genetic changes introduce a premature stop codon, result in the production of a truncated non-functional protein (Figure 3). These mutations are usually associated with the severe infantile form, as the resulting protein is completely inactive [44]. Deletion or insertion mutations can occur, resulting in the loss or addition of nucleotides, altering the reading of the genetic code (frameshift) and producing non-functional proteins. For example, a mutation of c.525delT results in a complete loss of function of the enzyme [45]. Mutations in splicing sites, where some variants can affect DNA sequences that regulate the way messenger RNA is processed, lead to abnormal transcripts of the *GAA* protein. These errors can significantly reduce the effectiveness of the enzyme [46] (Figure 3).

The relationship between the mutation type and the clinical severity of PD is not always linear, but some general trends have been observed. One example is the severe mutations (non-sense, frameshift) that tend to be associated with the infantile form of PD (IOPD). In these cases, the enzyme is absent or almost completely inactive, leading to severe symptoms from the earliest months of life. C.525delT and other deletions or frameshifts are common in infantile forms, where enzyme activity is reduced to very low levels or is absent. Less severe (missense) mutations may instead be associated with the late form (LOPD) [47]. In these patients, residual enzyme activity may be sufficient to delay the onset of symptoms until adulthood, with a slower course and less aggressive disease progression. The classic *GAA* gene mutation in LOPD is c.-32-13T>G, a common mutation in the Caucasian population [6]. This mutation alters messenger RNA splicing but does not completely abolish enzyme activity, allowing partial production of the enzyme. In conclusion, mutations in the *GAA* gene are the major cause of PD, directly affecting the amount and efficiency of the enzyme acid alpha-glucosidase, which is essential for glycogen degradation. The different types of mutations (missense, non-sense, deletions, insertions and splice-site mutations) lead to different degrees of impairment of enzyme activity, resulting in a wide range of clinical manifestations from the most severe childhood forms to the less aggressive late forms [48]. Understanding mutations in the *GAA* gene is therefore crucial for accurate diagnosis, individualized management and the development of future treatments for PD [36].

## 4. Multisystemic Disease: Approach to Treatment

The main characteristic of PD is that it affects multiple systems [49]. Patients may experience symptoms affecting different systems [7,50,51,52]. The skeletal muscles are most affected [2]. The main symptom is myopathy, with progressive muscle weakness [1]. In the infantile form, the weakness is often severe and leads to motor difficulties from the first few months of life. In adult patients, the progression is slower but can still lead to significant loss of mobility. Cardiac involvement is particularly important, as cardiac hypertrophy can lead to heart failure and early death if left untreated. In adult patients, cardiac involvement is less common but not completely absent.

Another system that is affected is the respiratory muscle system. Respiratory muscle weakness is another serious complication of PD, often responsible for morbidity in adult patients [53]. This condition can lead to respiratory failure, requiring the use of assisted ventilation [54]. Less common, but not completely absent, is the involvement of the nervous system. There is some evidence that the central nervous system may be affected, particularly in patients with the infantile form. Neuronal damage may contribute to motor and cognitive dysfunction [55]. Other scientific research has focused on studying the gastrointestinal system in PD [56]. The accumulation of glycogen in the smooth muscle of the intestines can lead to digestive problems and difficulties in absorbing nutrients. For this reason, the scientific community is studying the extent and effects of ERT in PD. In particular, Aditi Korlimarla’s group studied the histopathology of the entire gastrointestinal tract in mice [56]. According to the study, gastrointestinal manifestations represent a significant burden for adults with LOPD and should be assessed during routine clinical visits using quantitative tools (PROMIS-GI measures). The study also highlights the need for next-generation therapies for PD that target smooth muscle. Furthermore, Harrison N. Jones et al. (2020) report tongue muscle weakness in patients with LOPD. To investigate the diagnostic potential of tongue involvement in LOPD, they conducted an assessment of tongue structure and function in 70 subjects, comprising 10 individuals with untreated LOPD, 30 individuals with other acquired or hereditary myopathies and 30 controls with neuropathy. Tongue strength was evaluated through manual and quantitative muscle testing [57]. In his work, Chafic Karam draws attention to the presence of abnormalities in the tongue, as observed on brain magnetic resonance imaging (MRI). Such abnormalities are typically not documented by the radiologist. A meticulous examination of the tongue during a brain MRI scan can prove invaluable in establishing an accurate diagnosis of muscle weakness in patients [58]. For this reason, the management of PD requires a multidisciplinary approach, involving the input of neurologists, cardiologists, pulmonologists, physical therapists and other specialists. Such a team can monitor disease progression and optimize treatment based on clinical changes [59].

An early diagnosis of PD is important because early treatment can slow the progression of the disease and improve patients’ quality of life. It is also helpful to start treatment before irreversible damage occurs to the muscles and organs affected by the disease. ERT is currently the standard treatment for PD [60,61]. However, response to ERT can vary widely between patients due to genetic and immunological differences. Precision medicine aims to tailor therapy to a patient’s genetic profile, optimizing the dose and frequency of treatment to maximize efficacy and minimize side effects. In addition, gene therapies are being developed that may provide more durable genetic correction than ERT. Research into ERT for lysosomal storage disorders dates back more than four decades to the 1980s [62]. A major development in this field was the introduction of the first ERT for Gaucher disease, a condition characterized by abnormal accumulation of material within the lysosomes. The crucial importance of oligosaccharides was highlighted, as they play an essential role in directing enzymes to specific receptors for endocytosis, the mechanism by which substances enter cells. However, while therapy for Gaucher disease has shown some success, tackling PD with ERT has proven to be a more daunting task. Despite these challenges, the attempt to treat PD has led to fundamental discoveries. It was recognized that exogenous enzymes, once introduced into the body, could be internalized by lysosomes via mannose-6-phosphate (M6P) receptors, thereby harnessing natural cellular mechanisms to exert their therapeutic function. This understanding paved the way for the development of glucosidase alpha, the first treatment to directly target the primary cause of PD [63,64]. This exogenous enzyme is designed to supplement or replace deficient enzyme activity in patients, thereby significantly improving disease management. In 2006, glucosidase alpha was approved for commercial use worldwide, marking a historic breakthrough in the treatment of this disease in both the US and Europe [65,66]. This approval was a major milestone, offering new hope to patients suffering from a previously difficult-to-treat disease [66]. The culmination of decades of research into the structure, function and processing of acid alpha-glucosidase was the approval of glucosidase alpha (a recombinant version of human acid alpha-glucosidase, marketed as Myozyme^®^/Lumizyme^®^) in 2006. This was the first enzyme replacement therapy to become available for PD [66]. This treatment proved to be a lifesaver for patients who had been diagnosed with the disease in childhood, while also significantly improving the quality of life for those who had been diagnosed with the disease in adulthood. However, the long-term clinical experience has revealed certain unmet needs. Despite the administration of therapy, the progression of PD persists, particularly within skeletal muscles, which results in functional limitations. The close collaboration between the scientific community and patients has resulted in the enhanced awareness of the disease, a more comprehensive understanding of its pathophysiology and clinical course in patients beyond the first decade of life and a more nuanced appreciation of the strengths and limitations of enzyme replacement therapy. These advances have stimulated the development of a new generation of therapies and provided a solid basis for the introduction of new treatments.

## 5. Epigenetics

Precision medicine is based on the uniqueness of each patient and understanding that genetic, epigenetic and environmental variables can lead to more effective and targeted treatments [67]. Epigenetics refers to changes in gene expression that do not involve changes in the DNA sequence itself. These changes can affect how genes are switched on or off and are often regulated by mechanisms such as DNA methylation, histone modification and the action of microRNAs [68]. In PD, research into epigenetics is still in its early stages, but there are some areas of interest, e.g., the regulation of *GAA* gene expression. For example, methylation of the *GAA* gene promoter could reduce the expression of the enzyme acid alpha-glucosidase, making the disease worse. The response to ERT is subject to considerable inter-individual variability, and it may be hypothesized that epigenetic factors exert an influence on this response. For instance, epigenetic modifications may affect the absorption or distribution of the administered enzyme.

Another factor could be microRNAs. MicroRNAs (miRNAs) are small non-coding RNAs that regulate post-transcriptional gene expression. Some studies have investigated the role of miRNAs in PD, suggesting that they may influence glycogen degradation processes or the inflammatory response associated with the disease [69]. Shahzeb Hassan et al. reviewed three common epigenetic mechanisms—DNA methylation, histone modifications and microRNAs—and highlighted their applications to phenotypic variation and therapeutics [67]. A preliminary analysis of the plasma of six patients was conducted by Tarallo, revealing 55 microRNAs that exhibited differential expression. Sixteen of these microRNAs were found to be relevant to the pathophysiology of PD, particularly with regard to autophagy, muscle regeneration and muscle atrophy. One of these microRNAs, namely miR-133a, was selected for further quantitative analysis by real-time polymerase chain reaction in plasma samples from 52 patients, obtained from seven Italian and Dutch biobanks. Levels of miR-133a were found to be significantly higher in patients with PD, which could assist clinicians in initiating specific investigations for the diagnosis of PD [70].

Understanding the epigenetic mechanisms involved in PD could lead to the development of new targeted therapies, e.g., drugs that modify DNA methylation or histone acetylation could potentially modulate *GAA* gene expression or improve the efficacy of enzyme replacement therapy. In summary, although research into epigenetics in PD is still at an early stage, it is a promising area of study that could improve our understanding of the disease and open up new therapeutic avenues. Further investigation into genetic modifiers, including epigenetic factors, will be crucial for personalizing treatment and improving outcomes for individuals with PD and other lysosomal storage disorders

## 6. Evolving Scenarios and Motor Function Assessment: Outcome Measures Relevant to Vital, Cognitive and Neurological Status

The assessment of motor function and outcome measures relevant to vital, cognitive and neurological status in PD are key aspects of the clinical management of this rare disease [68]. The assessment of motor function is essential for monitoring the progression of muscle weakness, which mainly affects skeletal muscles and, in severe cases, respiratory muscles [71]. Tools used by clinicians to date may include tests such as the Medical Research Council (MRC) muscle strength scale, assessment of walking ability (6 min walk test), assessment of fine and gross motor skills and tests of respiratory function (vital capacity, respiratory muscle strength) [72]. The main outcome measures considered include the monitoring of parameters such as respiratory function (very important in PD patients) and swallowing ability. In severe cases, respiratory impairment may require the use of assisted ventilation [57]. Although PD mainly affects muscles, there are rare cases of central nervous system involvement (especially in childhood forms), so monitoring of cognitive abilities may be relevant in some patients [72]. Neurological assessment focuses mainly on reflexes, coordination and any signs of progressive muscle weakness. In conclusion, the assessment of motor function and vital, cognitive and neurological status in PD aims to monitor disease progression and evaluate the effectiveness of treatments to improve patients’ quality of life and slow muscle and respiratory deterioration.

Furthermore, recent longitudinal studies utilizing brain magnetic resonance imaging (MRI) and cognitive and neuropsychological tests have revealed cerebral white matter abnormalities and varying degrees of cognitive decline in long-term survivors [55,70,73,74]. A recent publication also reported that, among a subset of IOPD patients on long-term ERT, central nervous system (CNS) manifestations, including hyperreflexia, encephalopathy and seizures, had become prominent. There was likely an association between these symptoms and significant white matter hyperintensities (WMHIs) on MRI [72].

## 7. Development of Informatic Platforms (IT): Application of Artificial Intelligence Algorithms Useful for Diagnosis of PD Patients

To optimize clinical management and improve the diagnosis and follow-up of patients with this disease, an integrated platform that can collect, analyze and interpret complex data from different sources, such as clinicians and geneticists, or from different departments, could be helpful. Some researchers are developing IT platforms that allow comprehensive management of PD data to improve early and accurate diagnosis, patient follow-up, identification of disease outcome measures and personalization of therapies [49,75]. Simon Lin’s group at the Science Department in Vienna conducted a study using Symptoma artificial intelligence for the systematic identification of patients with rare diseases and automated phenotyping specific to PD [75]. Retrospective electronic medical records were used, which are a rich source of health and condition data for all patients. This provided new insights into the characteristics of patients with suspected PD. The platform includes integration with wearable devices that collect real-time data on patient mobility, heart rate, respiratory capacity and other physiological parameters. These devices enable continuous monitoring, improving the quality of care and facilitating early intervention in the event of clinical deterioration. The project to develop an IT platform for integrated data management in PD would be a major step forward in identifying and personalizing treatment. By bringing together clinicians, geneticists, biomedical engineers and bioinformaticians, it is possible to improve the quality of diagnosis, monitoring and patient outcomes by using advanced technologies such as artificial intelligence and wearable smart devices.

Another study utilized electronic health records from the Abu Dhabi Healthcare Company (SEHA) healthcare network in the UAE to develop an expert rule-based screening approach operationalized through a dashboard [76]. The study encompassed six patients diagnosed with IOPD and involved the screening of 93,365 subjects. A set of expert-derived rules was formulated to identify potential high-risk IOPD patients based on their age, the presence of particular symptoms and creatine kinase levels. The proposed approach was evaluated in terms of its accuracy, sensitivity and specificity. The proposed approach demonstrated accurate identification of five true positives, one false negative and four false positive IOPD cases. In 2017, Hamed’s group published an observational study with a twofold objective: firstly, to acquire real-world data on mobility and daily activity through a commercially available wearable device in patients with LOPD; secondly, to explore the relationship between ERT infusion time, disease severity and activity measured by the device. The dataset comprised parameters such as step count, distance traveled and elevation, along with the correlation between ERT infusion time, disease severity and activity measured by the device. Secondly, the study sought to examine the rate of adoption and adherence to wearable technology among patients with LOPD. Participants were provided with Fitbit One devices for a period of six weeks to monitor activity [77].

The utilization of smart technology has the potential to facilitate novel solutions in the clinical management of patients, thereby enhancing the quality of care and research on neuromuscular disorders. Ricci and her research group have developed a mobile application, called AIGkit, specifically designed for adult patients with PD. The aim of the application is twofold: firstly, to help patients manage the burden of disease-related factors; secondly, to provide doctors with the continuous monitoring of each patient in real time and under the environmental conditions of daily life. The AIGkit is presented as an innovative approach that harnesses cutting-edge technology to improve the quality of care and research in neuromuscular disorders [78].

This multidisciplinary and technologically advanced approach paves the way for new models of rare disease management, with potential applications in other clinical areas.

## 8. Conclusions

In conclusion, this review emphasizes the critical need to fill existing gaps, such as the development of more reliable biomarkers, to achieve early diagnosis. By summarizing key findings and proposing innovative approaches, such as the integration of artificial intelligence with genetic tools, this work aims to provide a clear direction for future research. These insights not only highlight current challenges, but also inspire new avenues of exploration, fostering progress and collaboration in the search for new solutions. The results of this study also highlight the critical role of genetic technologies in the diagnosis and management of PD, emphasizing the necessity of these tools not only for the identification of *GAA* gene variants but also for the timeliness and personalization of therapeutic interventions. However, significant gaps remain in the ability to translate these findings into concrete clinical improvements, and an important challenge is also to search for useful tools to monitor disease progression or to evaluate the effectiveness of therapies. The integration of innovative approaches, such as the application of artificial intelligence to genetic and clinical data, could accelerate the discovery of new therapeutic targets and improve patient stratification. Future research directions should therefore focus on developing more sensitive and predictive diagnostic tools and exploring multidisciplinary methodologies combining genomics, proteomics and computational analysis.
